# Unravelling the Role of Gut and Oral Microbiota in the Pediatric Population with Type 1 Diabetes Mellitus

**DOI:** 10.3390/ijms251910611

**Published:** 2024-10-02

**Authors:** Stefania Luppi, Luana Aldegheri, Eros Azzalini, Emanuele Pacetti, Giulia Barucca Sebastiani, Carolina Fabiani, Antonietta Robino, Manola Comar

**Affiliations:** 1 Institute for Maternal and Child Health—IRCCS Burlo Garofolo, 65/1 Via dell’Istria, 34137 Trieste, Italy; stefania.luppi@burlo.trieste.it (S.L.); luana.aldegheri@burlo.trieste.it (L.A.); manola.comar@burlo.trieste.it (M.C.); 2 Department of Medicine, Surgery and Health Sciences, University of Trieste, Strada di Fiume 447, 34149 Trieste, Italy; eazzalini@units.it (E.A.); s297842@ds.units.it (E.P.); 3 Medicine of Services Department, Clinical Analysis Laboratory, Azienda Sanitaria Universitaria Giuliano Isontina, 34125 Trieste, Italy; giulia.baruccasebastiani@asugi.sanita.fvg.it (G.B.S.); carolina.fabiani@asugi.sanita.fvg.it (C.F.)

**Keywords:** T1DM, gut microbiome, oral microbiome, SCFAs, probiotics, FMT, children

## Abstract

Type 1 Diabetes Mellitus (T1DM) is a chronic autoimmune disease that results in the destruction of pancreatic β cells, leading to hyperglycaemia and the need for lifelong insulin therapy. Although genetic predisposition and environmental factors are considered key contributors to T1DM, the exact causes of the disease remain partially unclear. Recent evidence has focused on the relationship between the gut, the oral cavity, immune regulation, and systemic inflammation. In individuals with T1DM, changes in the gut and oral microbial composition are commonly observed, indicating that dysbiosis may contribute to immune dysregulation. Gut dysbiosis can influence the immune system through increased intestinal permeability, altered production of short chain fatty acids (SCFAs), and interactions with the mucosal immune system, potentially triggering the autoimmune response. Similarly, oral dysbiosis may contribute to the development of systemic inflammation and thus influence the progression of T1DM. A comprehensive understanding of these relationships is essential for the identification of biomarkers for early diagnosis and monitoring, as well as for the development of therapies aimed at restoring microbial balance. This review presents a synthesis of current research on the connection between T1DM and microbiome dysbiosis, with a focus on the gut and oral microbiomes in pediatric populations. It explores potential mechanisms by which microbial dysbiosis contributes to the pathogenesis of T1DM and examines the potential of microbiome-based therapies, including probiotics, prebiotics, synbiotics, and faecal microbiota transplantation (FMT). This complex relationship highlights the need for longitudinal studies to monitor microbiome changes over time, investigate causal relationships between specific microbial species and T1DM, and develop personalised medicine approaches.

## 1. Introduction

Type 1 Diabetes Mellitus (T1DM) is a chronic autoimmune disorder characterised by the destruction of insulin-producing β cells in the pancreas. This leads to hyperglycaemia and requires lifelong insulin therapy. The precise aetiology of T1DM is not yet fully elucidated. Nevertheless, it is widely acknowledged that genetic predisposition and environmental factors play an essential role in the development of the disease [1]. Recently, increased research has focused on the role of the gut and oral microbiomes in T1DM, given their significant influence on immune system regulation and systemic inflammation. Specifically, gut dysbiosis may result in increased intestinal permeability, altered production of short chain fatty acids (SCFAs), and abnormal immune responses, all of which may contribute to the pathogenesis of T1DM [2]. On the other hand, the oral microbiome, along with the gut microbiome, is becoming increasingly known for its crucial role in systemic health. The oral cavity serves as the primary gateway for pathogens and is essential for maintaining overall microbial homeostasis. Dysbiosis in the oral microbiome has been associated with the onset of chronic inflammation and periodontal disease, conditions that are particularly prevalent in individuals with T1DM. Moreover, periodontal disease can exacerbate systemic inflammation, thereby influencing the progression and management of T1DM [3]. 

Despite the mounting evidence of the important roles played by the gut and oral microbiomes in T1DM, the precise mechanisms linking microbial dysbiosis to the disease remain underexplored. It is extremely important to understand these relationships, as this may lead to the identification of novel biomarkers for early diagnosis and disease monitoring, as well as the development of targeted therapeutic interventions aimed at restoring healthy microbial communities.

The purpose of this review is to synthesize the existing research on the interplay between T1DM and microbiome dysbiosis, with a particular focus on the gut and oral microbiomes. By reviewing the current scientific literature, we aim to elucidate the mechanisms by which microbial imbalances may contribute to the pathogenesis of T1DM and to highlight the potential for microbiome-based therapies to improve disease outcomes. 

## 2. Type 1 Diabetes Mellitus

T1DM is one of the most prevalent endocrine and metabolic disorders of childhood [4]. The incidence of T1DM is on the rise globally, with a significant impact on global health expenditure, one which has been estimated at 760 billion USD in 2019. Annually, nearly 500,000 children are diagnosed with T1DM, and the global burden of T1DM is projected to reach 13.5–17.4 million by 2040 [5,6]. 

T1DM is characterised by a chronic hyperglycaemia and insulin deficiency due to the loss of pancreatic islet β cells [7,8]. β cell loss is usually attributed to T1DM-associated autoimmunity, resulting in the appearance of T1DM-associated autoantibodies many months or years before the onset of clinical disease, a state referred to as seropositivity. However, in 10–30% of patients, no autoantibodies are detected, and the cause of β cell destruction remains unknown. Autoantibodies characteristic of immune-mediated T1DM include GADA (glutamic acid decarboxylase autoantibody), IAA (insulin-directed autoantibody), IA2 (antibodies directed against tyrosine phosphatase), and ZNT8 (antibodies directed against zinc transporter 8) [9,10,11]. 

The diagnosis is usually based on the presence of classic T1DM symptoms, which include polyuria; polydipsia; weight loss and, in some cases, abdominal symptoms; headache; fatigue; and ketoacidosis. Today, people with this condition still experience significant morbidity and mortality due to chronic complications such as diabetic retinopathy, peripheral sensorimotor neuropathy, and other cardiovascular diseases including heart failure [1]. 

T1DM is a multifactorial disease involving both genetic and environmental factors. The most important genetic risk factors are the HLA class II haplotypes HLA-DR3-DQ2 and HLA-DR4-DQ8, but several studies have shown that many non-HLA genetic factors also contribute to T1DM predisposition [12]. 

In particular, the following genes have been identified as being associated with T1DM: PTPN22 (protein tyrosine phosphatase non-receptor type 22), INS (insulin), CTLA4 (cytotoxic T-lymphocyte-associated protein 4), and IL2RA (interleukin 2 receptor alpha) [13]. 

Given the rapid increase in T1DM incidence rates in recent years, particularly in high-income countries, and the fact that only 10% of individuals with a genetic predisposition will develop T1DM, environmental and lifestyle factors have also emerged as important contributors to the onset of the disease [14]. In particular, environmental factors contributing to T1DM include viral infections (e.g., rubella and enterovirus), diet and microbiota. With regard to the latter, studies have demonstrated the pivotal role of the gut microbiota in the development and maturation of the immune system, suggesting that gut dysbiosis may also result in impaired immune function [15]. This has led to the hypothesis that the gut microbiome may be involved in the development and progression of T1DM, and thus several efforts have been made to investigate this link. Moreover, recently, a possible role of the oral microbiome in T1DM is also emerging.

## 3. Gut and Oral Microbiota

The gut microbiota, which is the collective term for the system of bacteria, archaea, and eukarya colonizing the intestinal tract in a human adult, is estimated to comprise approximately 10^13^–10^14^ microorganisms living in symbiosis with the host. The gut microbiota is the largest of the human body and is commonly classified into six major phyla. Although the prevalence of each phylum varies along the length of the intestine and between the lumen and mucosa, the majority of the microbial population, representing approximately 90% of the total, comprises the Gram-negative Bacteroidetes and the Gram-positive Firmicutes. The remaining 10% is distributed among the Actinobacteria, Proteobacteria, Fusobacteria, and Verrucomicrobia [16].

In physiological conditions, the equilibrium of the intestinal tissue and the inflammatory response against these microorganisms, which contain a high concentration of molecules that are not self-derived, are finely tuned by different regulatory mechanisms. This regulation appears to be a process fundamental for the development and education of a functional immune system, particularly during the first few years of life. Alterations in the composition of the gut microbiota and subsequent imbalance of the microbial ecosystem, a condition termed dysbiosis, may result in a chronic inflammatory state and the development of different immune-mediated diseases, including T1DM [17].

The composition of the microbiota of each individual begins to take shape even before conception, when the microbiota of the father exerts an influence on the microbiota of the mother, and vice versa [18]. Moreover, recent studies have challenged the long-held belief that the uterus is sterile, suggesting that microbes may also be present in the placenta, amniotic fluid, fetal membrane, and umbilical cord blood [19,20]. Indeed, maternal vaginal infections or periodontitis can result in the transportation of vaginal and oral bacteria to the uterine tissue and subsequently to the fetus through the bloodstream [21]. 

The mode of delivery also affects the initial bacterial composition of the newborn. Vaginally delivered neonates exhibit a microbiota that is more closely aligned with the vaginal microbiome of the mother, characterised by the prevalence of *Lactobacillus*. In contrast, neonates delivered via cesarean section are colonized by skin and environmental microbes, including *Staphylococcus* and *Clostridium* genera [22]. During the first six months following birth, the faecal microbiota of the newborn is initially like that of the mother. However, as the infant approaches one year of age, the gut microbiota matures, shifting from a predominance of Actinobacteria and Proteobacteria toward a predominance of Firmicutes and Bacteroidetes. Overall, the maturation processes end during the third year of life, resulting in a microbiome composition more similar to that of an adult. During this period, postnatal factors, including antibiotic use, diet, the infant’s genetic makeup, and environmental exposure, exert an influence on the infant’s microbiome [23].. 

In addition to the gut microbiota, other microbiota can be identified in the human body, including those associated with the oral cavity, respiratory system, vagina, and skin [24]. Among these, the oral microbiota is the second-largest microbiota, consisting of approximately 700 species that colonize the teeth, mucosal surfaces, and saliva [25]. In general, the oral microbiota of healthy individuals is dominated by Actinobacteria (genera *Corynebacterium*, *Rothia*, and *Actinomyces*), Firmicutes (genus *Streptococcus*), Fusobacteria (genus *Fusobacterium*), and Proteobacteria (genera *Neisseria* and *Haemophilus*), as well as Bacteroidetes (genera *Prevotella* and *Porphyromonas*) [26]. However, significant variations in the composition of the microbiota can be observed between different oral niches, life-history stages, and dietary habits [27]. For instance, the tooth surface is commonly colonized by *Streptococcus* spp. and anaerobic bacteria such as *Fusobacterium*, *Veillonella*, and *Actinomyces*, while mucosal tissue is prevalently colonized by *Streptococcus* spp. [28,29]. In contrast, the composition of the saliva microbiota is highly variable, encompassing microorganisms from all the different oral niches. Different authors have reported that the most dominant phyla in the oral cavity are Proteobacteria, Firmicutes, Actinobacteria, Fusobacteria, and Bacteroidetes. At the genus level, the most frequently reported microorganisms are *Streptococcus*, *Neisseria*, *Haemophilus*, *Prevotella*, *Veillonella*, *Rothia*, *Granulicatella*, and *Fusobacterium*. However, the data on the literature are highly variable, and further studies are needed to better define a core salivary microbiota [30,31,32]. The fact that 0.75–1 L of saliva are ingested every day by the gut has prompted the scientific community to investigate the mutual influence between the two compartments with respect to their microbiota composition. Although gastric acid and bile can act as a bottleneck in the selection for specific bacteria, recent evidence has demonstrated how effectively oral bacteria can translocate to the gut and modulate gut microbiota [33,34]. This intimate interconnection between the two compartments may play an important role in the development and progression of several disorders, including gastritis, inflammatory bowel disease, colorectal cancer and T1DM [3]. For example, Schmidt and colleagues demonstrated that the majority of oral species are transferable to the gut, with increased levels of transmission observed in patients with colorectal cancer and rheumatoid arthritis, compared to healthy subjects [35].

## 4. Gut Microbiome in T1DM

Although a causal link between microbiome changes and T1DM has been established in experimental models, their relationship remains a challenge in humans [36,37]. This is due to several factors, including the complexity of the interactions between the microbiota and the immune system and the lack of placebo-controlled studies able to follow long-term changes in the gut microbiome and the associated immune response in relation to T1DM outcomes. In addition, both the clinical onset of T1DM and seroconversion typically occur within the first years of life, a period during which the gut microbiome undergoes significant changes driven by diet, geographic location, mode of birth, infections, and antibiotic treatments.

However, several studies have been conducted to investigate whether variations in the gut microbiome are associated with T1DM, looking at people at high genetic risk and at different stages of the disease, including seroconverted patients and those with clinical T1DM.

Gut dysbiosis and the subsequent impairment of gut permeability have been identified as likely disruptors of immune tolerance that may lead to T1DM [38,39], suggesting that subjects with T1DM are indeed “leaky gut” patients [40,41]. The gut barrier is a complex structure comprising gut microbiota, mucus, enterocytes, tight junction (TJ) proteins, and innate and adaptive immune cells [42]. One of the potential pathways through which gut microbiota affect intestinal permeability appears to be dependent on high levels of zonulin, the production of which can be influenced by bacterial colonization. Zonulin has been identified as a regulator of intestinal permeability, exerting its effect by modulating TJs. It has been demonstrated that an increase in zonulin release occurred concomitant with an elevation in permeability prior to the manifestation of clinically apparent T1DM. It was hypothesised that an imbalance in microflora colonization could induce the upregulation of zonulin within the gut lumen. The released zonulin was then recognised by receptors on the surface of intestinal epithelial cells, resulting in alterations to TJ dynamics, including cytoskeletal remodelling and the phosphorylation of zonula occludens-1 and occludin. Ultimately, this led to an enhancement in gut permeability due to the disassembly of TJs [2,43]. The disruption of the integrity of the intestinal barrier may lead to the passage of infectious factors, food antigens, microbial antigens, and products of the microorganisms themselves such as bacterial endotoxin from the intestinal mucosa to the pancreatic lymph nodes, where they may trigger or exacerbate T1DM [44,45]. 

An additional hypothesis regarding the potential molecular mechanism by which bacteria may modulate intestinal barrier permeability in T1DM is that of bacterial antigen mimicry, based on the evidence that some gut bacteria express glutamic acid decarboxylase (GAD) and produce gamma-aminobutyric acid (GABA). The death of GABA-producing bacteria at the onset of T1D through viral- or antibiotic-mediated mechanisms may release bacterial GAD. The enzyme may act as an antigen, stimulating the maturation of submucosal T cells. This process could potentially result in the miseducation of the host immune system due to the similarity between bacterial GAD and human GAD65 and could provoke the immune system to attack and produce antibodies targeting human GAD65-expressing β cells [46].

Several authors reported a significant association between bacterial lipopolysaccharides (LPS) and T1DM. A case-control study showed that subjects with T1DM had higher circulating LPS than non-diabetics [47], while Yuan and colleagues found a significant abundance of genes related to LPS biosynthesis in T1DM patients compared to controls by functional profiling of the gut microbiota [48]. In addition, Vatanen and colleagues showed that *Bacteroides* species produce a type of LPS with immunoinhibitory properties that prevent early immune development and contribute to the advancement of T1DM in the microbiota of children from countries with high susceptibility to autoimmunity [49]. 

In particular, it was shown that LPS, which is secreted by Gram-negative bacteria, can bind to toll-like receptor 4 (TLR4) on immune cells, thereby promoting the production of pro-inflammatory cytokines (e.g., IL-1β, IL-6, and TNF-α) and triggering immune activation. Chronic inflammation can thereby contribute to the damage of β cells and the development of autoimmunity [50].

Loss of gut microbiota diversity, which is one of the hallmarks of gut dysbiosis, has been identified in T1DM and other chronic diseases. Several studies have attempted to establish the validity of gut microbiome diversity as a biomarker for T1DM in humans, with mixed results. Recent evidence suggests a marked loss of α-diversity, β-diversity, and community richness in T1DM patients compared to healthy controls [51,52]. The loss of microbiota diversity was shown to follow a temporal trend and to be age-related [53]. Specifically, microbiota diversity decreases over time in autoantibody-positive children compared to autoantibody-negative children [54]. Furthermore, this decrease precedes the onset of the disease, making it an interesting biomarker for monitoring patients at different stages of progression. A concomitant enrichment in metabolic pathways associated with a pro-inflammatory environment, another marker of gut dysbiosis, was also detected in the time frame between seroconversion and T1DM onset, when community diversity decreases, strengthening the link between gut microbiome alteration and the development of T1DM [55]. Despite the presence of a body of evidence in the literature, it is worth noting that several studies failed to find an association between gut microbiota diversity and T1DM [56]. Differences in analytical methods and cohort selection may account for these discrepancies. Moreover, geographical location influencing gut microbial diversity, as well as HLA genotype, may be sources of bias that should be considered in clinical trials [57,58,59].

Another common feature reported by numerous authors in subjects with T1DM compared to healthy controls is a decrease in the Firmicutes (Gram-positive) to Bacteroidetes (Gram-negative) ratio (F/B). There is evidence to suggest that the F/B ratio decreases significantly over time in children with clinical T1DM or those who eventually progress to clinical T1DM, whereas it increases in non-diabetic children [51,58,60]. Moreover, the F/B ratio appears to be associated with the number of autoantibodies detected in patients [61]. Bacteroidetes and Firmicutes are among the major contributors to the biosynthesis of LPS and butyrate, respectively [62,63]. Therefore, an imbalance towards a higher abundance of Bacteroidetes may explain the higher levels of LPS and the concomitant alteration of SCFAs and gut permeability often observed in T1DM patients [64,65]. Nevertheless, several authors have reported no difference in the F/B ratio between healthy and T1DM individuals. Further studies are therefore required to clarify this relationship [66]. 

Despite documented differences in gut microbiota composition between healthy subjects, autoantibody-positive children, and children with clinical T1DM, there is no consensus in the literature on a microbial signature that can differentiate between these groups. Overall, genera such as *Bacteroides*, *Clostridium, Veillonella, Ruminococcus, Escherichia, Streptococcus, Enterobacter*, and *Lactobacillus* were frequently reported to be more abundant in T1DM patients whereas *Prevotella, Akkermansia, Bifidobacterium*, and butyrate-producing genera such as *Roseburia, Faecalibacterium,* and *Eubacterium* were more abundant in healthy subjects [52,60,67,68]. Among the T1DM-associated genera, *Bacteroides* is the most frequently reported in the literature. Several *Bacteroides* species were found to be enriched in the gut microbiome of children who eventually developed T1DM over time, including *Bacteroides ovatus*, *Bacteroides thetaiotaomicron*, and *Bacteroides uniformis*, while other species, such as *Bacteroides fragilis* and *Bacteroides vulgatus*, were reported to be significantly enriched in healthy controls [53].

A Finnish study of children at high genetic risk of developing T1DM found that *Bacteroides dorei* and *vulgatus* were highly abundant in seroconverted T1DM patients; interestingly, the peak of abundance of *Bacteroides dorei* was detected just before the appearance of the first autoantibody in the group of seroconverted patients, supporting the hypothesis that gut dysbiosis may predict T1DM in high-risk individuals [69]. In this context, Matos and colleagues observed the ability of some *Bacteroides dorei* strains isolated from T1DM patients to invade and damage the TJs of intestinal epithelial cells in vitro, a mechanism that may lead to the impairment of intestinal permeability and the disruption of the immune tolerance [70].

As mentioned above, although microbial targets at the genus or species level have been implicated in the onset and development of T1DM, recent metagenomic analyses suggest that microbial factors associated with seroconversion or T1DM onset are more likely to be functional and/or metabolic changes rather than changes in specific microbial taxa. Healthy individuals were found to have a more functionally diverse microbiome compared to those with diabetes [67,71]. Accordingly, one of the largest longitudinal analyses on T1DM, the TEDDY study, did not find any significant taxonomic differences in the gut microbiota of seroconverted children or those with clinical disease compared to controls; on the other hand, in the same study a significant reduction in SCFA-expressing genes was detected in T1DM patients by metagenomic analysis [72]. Similar results were reported by Goffau and colleagues, who observed a drastic reduction in butyrate-producing species in the time frame between patient’s seroconversion and clinical onset of the disease [54]. 

Given the complexity of the gut microbiome and its functional network, machine learning and functional metagenomic analyses were also employed to identify significant taxa and pathways associated with T1DM or relevant to the metabolic control management of newly diagnosed children [51,73,74,75]. 

For instance, the relative abundance of *Bacteroides stercoris* was significantly higher in a cohort of Italian children with T1DM who also exhibited impairment in carbon, sugar, and iron-related pathways [76]. Tan et al. combined a deep learning and multi-omics approach and demonstrated that T1DM patients are associated with altered microbiota and lipidomic signatures. Notably, machine learning approaches utilizing the microbiota composition demonstrated inferior diagnostic performance compared to those utilizing metabolite composition to identify T1DM patients [77].

More recently, Yuan and colleagues conducted an integrative profiling of gut microbial functional and metabolic changes in children with new-onset T1DM in two different cohorts. Their multi-omics analyses demonstrated that LPS biosynthesis is increased in T1DM-associated gut dysbiosis, while butyrate production and bile acid metabolism exhibited an inverse trend. Furthermore, the combination of nine bacterial species and nine faecal metabolites was found to be able to predict new-onset T1DM [48]. In another study of adolescents with T1DM, patients exhibited alterations in nineteen microbial metabolic pathways related to fermentation and vitamin biosynthesis (B2/flavin, B7). The study also revealed that the taxonomic composition of the gut microbiome exhibited only modest changes in comparison to healthy subjects. These changes were observed in the context of alterations in microbial metabolic pathways related to fermentation, vitamin biosynthesis (B2/flavin, B7), enzyme cofactors (NAD+ and s-adenosylmethionine), and amino acids (aspartate, asparagine, and lysine). Supervised modeling identified *Coprococcus* and *Streptococcus* taxa as being the most predictive of T1DM status. It is important to note, however, that the bacterial species associated with dietary and clinical factors, including HbA1c, BMI, and daily intake of dietary nutrients and phytochemicals, differed between healthy adolescents and adolescents with T1DM. For example, the genus *Christensenella* was found to be negatively associated with dietary fat and protein intake in healthy subjects, but not in individuals with T1DM. In contrast, bacteria belonging to the *Bacteroidales* order were observed to be negatively associated with soluble fiber intake in children with T1DM, but not in healthy subjects [78]. This suggests that diet may have a notable impact in defining microbiome composition and risk of developing T1DM. To this merit, Endesfelder and colleagues identified the presence of three bacterial communities prior to the development of islet autoantibodies, which were found to be functionally associated with diet. Two communities, designated as C1 and C2, exhibited a pronounced association with breastfeeding and the introduction of solid foods, respectively, whereas no discernible dietary pattern was identified in the third community, C3. Nevertheless, C3 encompassed a subset of children who had initiated the non-milk diet at an early stage, particularly meat, and were dominated by *Bacteroides*. Notably, this subgroup exhibited an elevated risk for early autoantibody development [79].

**Figure 1:**
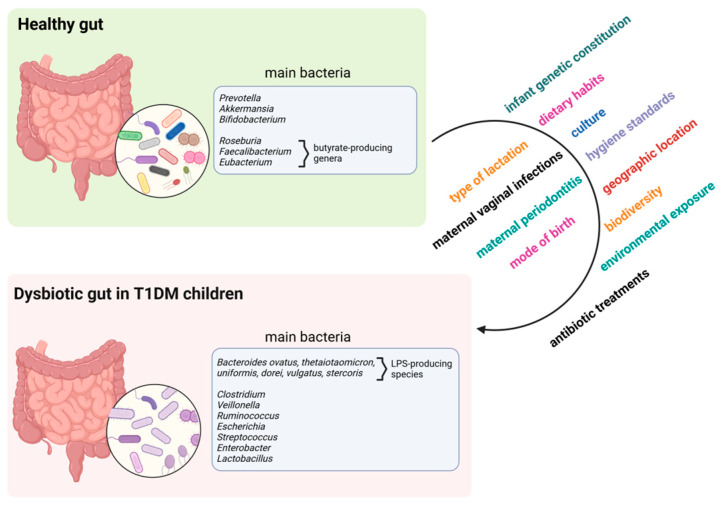
Potential contributors to the alteration of gut microbiome from a healthy to a dysbiotic one in T1DM children. Created with BioRender www.biorender.com (2 September 2024).

The main bacteria associated with healthy or dysbiotic gut in T1DM children are depicted in Figure 1. Moreover, a summary description of these variations is shown in Table 1.

## 5. SCFAs in T1DM 

With consideration of the intestinal microbiota, evidence indicates that SCFAs may exert a potential influence on the aetiology of T1DM. SCFAs are metabolites made up of less than six carbon atoms. The most important are acetate, propionate, and butyrate, which account for about 90–95% of total SCFAs. Although these are the predominant ones, isobutyrate, isovalerate, valerate, isocaproate, and caproate are also present in smaller quantities. They are produced primarily by bacterial fermentation of complex, indigestible polysaccharides, such as fiber, in the anaerobic environment of the cecum and colon. The production of these bioactive metabolites is influenced by several factors, such as the source of the substrate, the type and structure of the diet, the composition of the intestinal microbes, the pH value of the colon, the intestinal transit time, and the fermentation site of the substrate [80,81]. This highlights the intricate interplay between diet, gut microbiota, and SCFA metabolism. It is thus essential to maintain a state of intestinal eubiosis in order to prevent an altered production of these metabolites at this level and the subsequent adverse effects on the host’s health. SCFAs have been demonstrated to exert a range of biological effects on the host, including anti-inflammatory, immunoregulatory, anti-obesity, anti-diabetes, anticancer, cardiovascular-protective, hepatoprotective, and neuroprotective effects, some of which will be discussed in this chapter, as they are of particular interest with regard to T1DM [62]. 

T1DM individuals are known to exhibit an aberrant metabolic profile. Specifically, Winther et al. found that the levels of faecal propionate and butyrate were significantly lower in individuals with T1DM compared to healthy controls. The study reported median propionate levels of 9 µmol/g (range: 7.0–12) and butyrate levels of 7.8 µmol/g (range: 4.7–12) in the T1DM group. In contrast, the control group had higher median values of 11 µmol/g (range: 9–15) for propionate and 11 µmol/g (range: 8.1–16) for butyrate [82]. 

Butyrate is one of the most relevant intestinal SCFA and its production starts with the intake of dietary fibers. Fibers cannot be digested by human organism, so they reach the colon largely intact and become substrates for microbial fermentation from various anaerobic species, producing precisely SCFAs, gases, and other metabolites. The key bacteria involved in butyrate production belong to several genera, including *Faecalibacterium* (e.g., *Faecalibacterium prausnitzii*), *Roseburia*, *Eubacterium*, and *Anaerostipes* [83].

Within the intestine, butyrate-producing microbial communities act as “gatekeepers” regulating the influx of pathogenic bacteria across the intestinal barrier. In particular the presence of such bacterial species promotes an anaerobic environment in the intestine, preventing the colonization of opportunistic aerobic pathogens such as *Salmonella* and *E. coli*. In addition to its antimicrobial function, butyrate is involved in several other processes beneficial for the body [84].

Additionally, butyrate effectively regulates the integrity of the intestinal barrier by promoting the overexpression of TJ proteins, such as claudin-1, zonula occludens-1, and occludin [85]. Moreover, butyrate can strengthen the mucus layer of the gut epithelium by increasing the expression of mucin 2 [62,86]. An intact intestinal barrier acts as a shield against the translocation of pathogenic bacteria and antimicrobial peptides (AMPs) from the intestinal tract into the circulation, preventing local and potentially systemic inflammation and the initiation of autoimmune processes which can lead to T1DM.

Butyrate also exerts anti-inflammatory effects. In particular, it facilitates the differentiation of regulatory T cells within the immune system, which are critical in promoting immune tolerance and suppressing inflammatory and autoimmune responses [87]. In accord with this, FOXP3+ regulatory T-cells have been found to be decreased in T1DM patients [88].

In consideration of the previously outlined properties of butyrate, it can be said that dysbiosis and low SCFAs production may have implications for T1DM. Therefore, butyrate, through its activity on intestinal permeability, may play an important role in the progression of T1DM. However, the consequences of intestinal permeability and the cascade of molecular events that lead to the progression of T1DM are just beginning to emerge. A number of studies have provided convincing evidence that microbial SCFAs play a central role in the development of T1DM. One of the largest multicentric longitudinal studies on T1DM, the TEDDY study, demonstrated that the expression of microbial genes that regulate the biosynthesis of SCFAs was lower in children who developed T1DM than in matched controls [72]. Similarly, further evidence for this observation is provided by the longitudinal TRIGR and FINDIA studies conducted by de Goffeau and colleagues, who demonstrated that children with β cell autoantibodies exhibited a low abundance of lactate- and butyrate-producing gut microbiota (including species *Bifidobacterium adolescentis*, *Roseburia faecis* and *Faecalibacterium prausnitzii*) [54], indicating that alteration of the production of SCFAs represents an early event in the development of T1DM. It is noteworthy that the reduction of SCFAs may not be a universal phenomenon among all forms of diabetes, but rather a distinctive characteristic of T1DM. Hu et al. compared patients with type 2 diabetes mellitus (T2DM) and those with adult-onset T1DM and found that patients with adult-onset T1DM exhibited a notable depletion of SCFA-producing bacteria, particularly *Eubacterium rectale*. This was associated with a significant depletion of phenolic acids and their derivatives, including 3,4-dihydroxyhydrocinnamic acid, which has been linked to pancreatic β cell autoimmunity [89]. 

One of the key aspects of butyrate’s influence on T1DM is its ability to modulate the immune system. In particular, the immune system mistakenly targets and destroys pancreatic β cells. In a recent comprehensive investigation, Lo Conte and colleagues examined the interplay between gut barrier integrity, immunological status of the intestinal tissue, and the composition of the mucus-associated gut microbiota in patients with T1DM and healthy controls. The results demonstrated a notable impairment of the gut barrier in T1DM patients, accompanied by a reduction in mRNA expression of various mucins. It is noteworthy that alterations in the mucus layer were found to correlate with a reduction in the relative abundance of SCFA-producing bacteria, which regulate mucin expression and intestinal immune homeostasis. These bacteria include *Bifidobacterium dentium*, *Clostridium butyricum* and *Roseburia intestinalis*. Furthermore, in individuals with T1DM, an imbalance was observed in the intestinal immune system, as evidenced by the elevated proportions of effector T cells, including T helper (Th) 1, Th17, and TNF-α+ T cells [65].

In line with this, Bell et al. demonstrated that the administration of a dietary supplement, designated HAMSAB (high-amylose maize-resistant starch modified with acetate and butyrate), led to an improvement in glycemic control in adults with long-standing T1DM, as evidenced by a reduction in glycemic levels after a six-week period. The increased SCFAs following the HAMSAB supplement resulted in modulation of gut microbiota function and composition, with an expansion of bacteria able to utilise the delivered supplement. Furthermore, a change in the composition of T cells, B cells, dendritic cells, and monocytes towards a more regulatory immune phenotype was also observed, thereby reinforcing the link between SCFAs production and immunomodulation [90].

Butyrate has been shown to promote the development and function of regulatory T cells (Tregs), which are essential for maintaining immune tolerance and preventing autoimmune responses. By enhancing Treg activity, butyrate may mitigate autoimmune attack on pancreatic β cells [91]. The link between butyrate and T1DM opens up potential therapeutic avenues. Dietary interventions aimed at increasing butyrate production, such as a high-fiber diet or supplementation with butyrate-producing probiotics, could be explored as strategies to prevent or manage T1DM in children. However, more research is needed to fully understand the optimal ways to employ butyrate as a therapeutic agent and to demonstrate its efficacy and safety in humans. 

## 6. Oral Microbiome in T1DM

In addition to the gut microbiome, emerging evidence has also established a correlation between the oral microbiome and several systemic diseases, including diabetes [92]. As several years may elapse between the initial β cell damage and the manifestation of clinical diabetes [93], studies have suggested that early diagnosis of diabetes by analyzing the oral microbiota could potentially allow early treatment and delay the development of T1DM in children with β cell autoimmunity. Indeed, patients with T1DM typically present with dry mouth symptoms, oral acidosis, and periodontal disease, which is significantly associated with longer duration of diabetes and poor glycemic control [94,95]. 

T1DM often leads to dehydration due to hyperglycemia, which results in reduced salivary flow and dry mouth. Saliva has a crucial role in controlling oral bacteria, maintaining pH balance, and protecting tissues from microbial overgrowth. In the absence of sufficient saliva, bacteria can proliferate uncontrollably, since saliva contains antimicrobial proteins like lysozyme, lactoferrin, and defensins which regulate microbial populations. If saliva is reduced, defence mechanisms are disrupted and pathogenic and acidogenic bacteria such as *Streptococcus mutans* and *Lactobacillus* can proliferate, further disrupting oral health and leading to conditions like oral acidosis and tooth decay [96].

Periodontal disease is one of the most common oral complications in T1DM and is closely linked to bacterial infection and chronic inflammation. The oral microbiota of patients with T1DM often shows a significant shift in microbial composition, with an increase in periodontopathogenic bacteria like *Porphyromonas gingivalis* and *Aggregatibacter actinomycetemcomitans*. These bacteria promote chronic inflammation in the gums mediated by LPS and TLR-4 interaction by triggering an immune response which can lead to damage in periodontal tissues, gingivitis, periodontitis, and, eventually, tooth loss if untreated [97]. 

Some authors have shown that hyperglycemia in T1DM accelerates the formation of advanced glycation end products (AGEs), which accumulate in periodontal tissues and interact with the receptor RAGE on immune and endothelial cells, enhancing the inflammatory response and promoting tissue destruction. Specifically, the AGE–RAGE interaction has been demonstrated to induce oxidative stress and activate signalling pathways such as NF-κB, which ultimately results in the release of pro-inflammatory cytokines (e.g., TNF-α, IL-6) and matrix metalloproteinases (MMPs). The oral biofilm also contributes to this process by stimulating the production of additional cytokines and matrix metalloproteinases (MMPs) which facilitate the degradation of connective tissue and bone in the periodontium, ultimately leading to periodontal destruction [98].

It is evident that prolonged hyperglycemia in T1DM children leads to an increased level of glucose in the saliva, providing more substrates for bacterial fermentation. Chen and colleagues demonstrated that oral dysbiosis in T1DM patients results in a shift from neutral or mildly acidic bacteria to acidogenic bacteria that dominate the oral microbiome. Bacteria such as *Streptococcus mutans*, *Lactobacillus*, and *Actinomyces* flourish in the elevated-glucose milieu that is characteristic of diabetes and, through fermentation, erode enamel and disrupt the oral pH balance, thereby exacerbating acidosis and increasing susceptibility to dental caries [99]. 

Although the oral microbiome in T1DM can be shaped by a variety of factors including family history of hyperlipidemia and periodontal health [100], distinct oral microbiome signatures characteristic of T1DM patients have been proposed. In a Dutch study that included 53 children with T1DM and 50 healthy controls, the taxonomic profiles of T1DM subjects showed a significantly higher abundance of taxa belonging to the phyla Actinobacteria and Firmicutes, including *Streptococcus* spp., *Actinomyces* spp., and *Rothia* spp., whereas the taxonomic profiles of controls were enriched in taxa belonging to the phyla Bacteroidetes and Proteobacteria, including *Pasteurellaceae* [101]. A growing body of literature indicates that the genus *Streptococcus* is significantly more abundant in the oral cavity of individuals diagnosed with T1DM [102,103,104], especially those with poor glycemic control [105,106]. A number of *Streptococcus* species have been identified as being able to reduce the pH of glucose broth; these include *S. mitis*, *S. oralis*, *S. anginosus*, and *S. gordonii*. It has been found that these species are significantly enriched in subjects with T1DM. Furthermore, the presence of cariogenic species, such as *S. mutans*, can facilitate the growth of pathogenic biofilms, thereby increasing the susceptibility to periodontal disease, a feature commonly observed in T1DM patients [106,107].

Yuan et al. found that the acute phase of children with T1DM was characterised by oral microbiota dysbiosis, which could be partially ameliorated by glycemic control. Compared to healthy children, the acute phase group was characterised by reduced microbial diversity and a higher prevalence of opportunistic pathogens, including *Streptococcus*, *Granulicatella*, *Rothia*, and *Rhodococcus*. In contrast, the genera *Veillonella* and *Prevotella* exhibited an opposite trend. Notably, T1DM-related genera were also associated with blood HbA1c, FBG (Fasting Blood Glucose), and WBC (White Blood Cells) levels [108].

By employing a multi-omic approach, Kunath et al. were able to ascertain a considerable prevalence of diverse taxa which are markedly linked to an acidic milieu within the oral cavity of T1DM patients. In particular, a higher abundance of the acid-tolerant pathogenic species *S. mutans* was observed in comparison to the acid-intolerant and commensal species *S. salivarius*. It is noteworthy that *S. mutans* was found to correlate with the expression of a bacteriocin, which suggests the existence of a competitive relationship between the two species [109]. In this context, also Moskovitz and colleagues demonstrated that the salivary microbiome of children with T1DM exhibited distinctive characteristics compared to that of the control group. In particular, the genus *Streptococcus* was found to be more abundant in subjects with T1DM, while the genus *Mogibacterium* was enriched in healthy subjects and correlated with salivary pH and the DMFT (Decayed, Missing and Filled Teeth) index. It is noteworthy that a considerable number of the identified bacteria were found to be associated with the gut microbiome in individuals with T1DM, which suggests the existence of a potential link between the two compartments [104]. 

**Figure 2:**
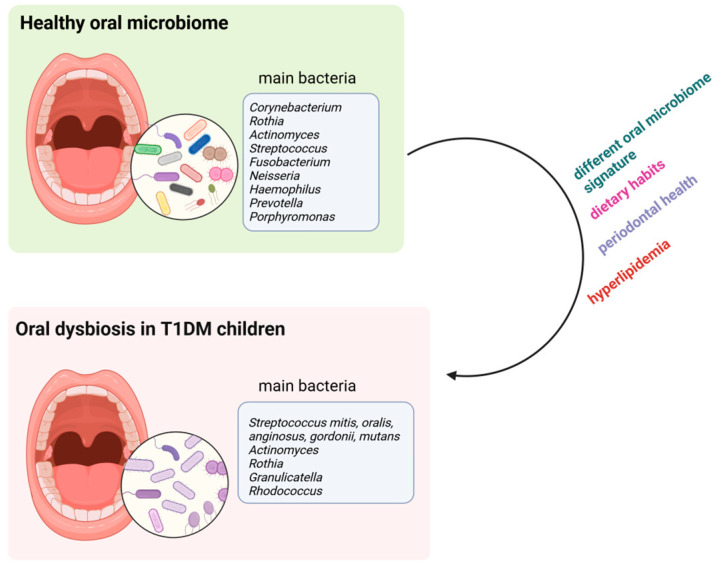
Potential contributors to the alteration of oral microbiome from a healthy to a dysbiotic one in T1DM children. Created with BioRender www.biorender.com (2 September 2024).

The main bacteria associated with healthy and dysbiotic oral environments in T1DM children are depicted in Figure 2. Moreover, a summary description of these variations is shown in Table 2.

## 7. The Interplay between Gut and Oral Microbiota in T1DM

**Figure 3:**
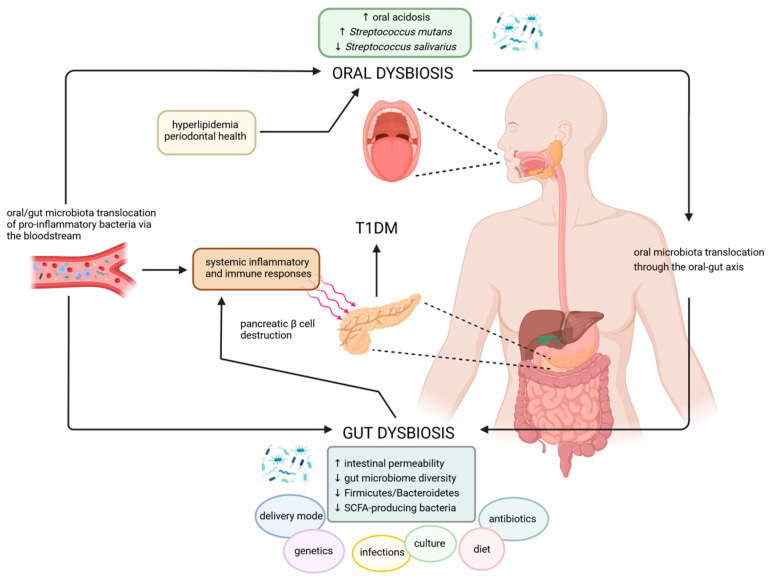
The interplay between gut and oral microbiota may contribute to the pathological processes of T1DM. Created with BioRender www.biorender.com (2 September 2024). **↑**—increase, **↓**—decrease.

Despite the fact that oral cavity and the gut are situated on opposite sides of the gastrointestinal tract, there is an increasing body of evidence to suggest that gut and oral dysbiosis may exert a mutual effect on each other and also at the systemic level [3,110,111]. Indeed, although gastric secretions exert a bactericidal effect between the oral and gut compartments, shared microbial species have been identified between the two sites [35,112]. Moreover, the microbial community present in the oral cavity, and in particular in the saliva, has been identified as a reliable predictor of the microbial community recovered from faeces. For example, individuals with a stool community type defined by the highest prevalence of *Prevotella* were found to be two-fold more likely to harbour saliva community types characterised by a high level of *Prevotella* [113]. In patients with T1DM, both oral and gut dysbiosis have been linked to poor glycemic control and systemic inflammation, indicating the presence of a complex regulatory network between the two sites. It is conceivable that oral and gut bacteria may contribute to the development of systemic disease by means of translocation through the oral–gut axis or by entering the circulatory system via periodontal tissue damage. Figure 3 presents a schematic illustration of the potential complex and significant interconnections between the gut microbiota and the oral microbiota in individuals with T1DM.

In a recent study, Yuan et al. conducted a comprehensive analysis of the blood, oral, and gut microbiome in 64 children with T1DM and 77 controls. The authors reported that the blood microbiome of T1DM patients exhibited partial overlap with the oral and gut microbiomes, indicating the potential for bacteria to be translocated from the gut and oral cavity to the bloodstream. Furthermore, a notable increase in the prevalence of pathogenic bacteria, including *Sphingomonas*, *Caulobacter*, and *Stenotrophomonas*, along with elevated inflammatory and glycolipid metabolism indicators, was observed in the blood of T1DM patients. Of particular significance was the finding that bacteria with the capacity to induce inflammation were more prone to enter the bloodstream in individuals with T1DM compared to controls, thereby indicating the involvement of the blood microbiome in the T1DM-associated inflammatory state [114]. In another study, Wang and colleagues examined the oropharyngeal and gut microbiome of 13 children with T1DM, identifying a potential correlation between the microbiota of these two sites. The authors posit that oral and intestinal pathogenic bacteria in children with T1DM may undergo synchronised alterations during disease states, a finding which could be harnessed for predicting disease trends. In particular, a strong correlation was identified between the abundance of oropharyngeal *Staphylococcus* and intestinal *Ruminococcaceae*, while oral *Streptococcus* exhibited a positive correlation with blood levels of C-peptide [115]. 

Similarly, Kunath et al. demonstrated that alterations in the oral microbiome also impacted the microbial communities and the inflammatory state in the lower gut, thereby reinforcing the interconnection between these two compartments in T1DM disease. In particular, a reduction in the number of *S. salivarius* bacteria in the oral cavity was associated with a reduction in the corresponding level in the gut and an increase in the number of *Enterobacteriaceae* bacteria, which are known to have the potential to cause disease. However, no correlation was observed between the metagenomic abundance of the taxa in the oral cavity and their transfer rate to the gut. This suggests that transfer rate may be influenced by additional factors, such as salivary flow, pH, or bacterial tolerance to bile acids [109]. 

Despite this evidence, research on the interplay between oral and gut microbiota in T1DM is still limited. Therefore, additional studies are needed to better understand the mechanisms that regulate the balance of oral and gut microbiota in T1DM. 

## 8. Possible Therapeutic Approaches

The modulation of the microbiome is one of the most hopeful new strategies in medicine relative to improving the health of diabetic patients [116]. The most promising therapeutic approaches involve the use of various nutritional factors which improve the composition and functionality of the gut microbiota, support glycemic control, reduce inflammation, and promote the general health and well-being of children affected by T1DM. Moreover, the development of novel targeted therapeutics such as faecal microbiota transplantation (FMT) has demonstrated favorable results in correcting microbial dysbiosis and restoring the immune state in the context of several diseases, including T1DM [117]. This approach, which targets alterations in the gut microbiota, has demonstrated favorable outcomes with notable advantages, including high clinical safety and minimal adverse reactions [118]. 

### 8.1. Prebiotics, Probiotics, and Synbiotics

Evidence has been reported that supplementation of prebiotics, probiotics, and synbiotics to drug therapy can reverse microbial dysbiosis in T1DM children, promoting an increase in health-related bacterial species and an elimination of pathogens, and thus a reduction of intestinal permeability and inflammation [119]. 

Prebiotics are selectively indigestible dietary components that have beneficial effects on the host by influencing colonic bacterial activity [120]. They are a potentially novel, inexpensive, low risk treatment supplementation for T1DM that may improve glycemic homeostasis, reduce intestinal permeability, and improve insulin sensitivity in T1DM children. Specifically, HMOs (human milk oligosaccharides) increase the abundance of *Bifidobacteria*, preventing islet autoimmunity and have been shown to be underrepresented in children with T1DM [121]. HAMS (high amylose maize starch) is a dietary fiber that can alter microbiome profile and metabolites. In a pilot randomized controlled trial, it was hypothesized that, as demonstrated in a rodent model, HAMS consumption shifts the gut microbiome profile towards dietary fiber fermenters producing abundant SCFAs (specifically, acetate and butyrate); thus, it was determined that acetylation and butyrylation of HAMS after colonic fermentation promote the release of large amounts of beneficial SCFAs, which can improve pancreatic β cell function, β cell health, and overall glycemia of youths newly diagnosed with T1DM [122]. In a pilot study on T1DM children, authors showed that the assumption of prebiotic oligofructose-enriched inulin for 12 weeks improves plasma C-peptide levels, and, consequently, children’s intestinal permeability, with a significant increase in the relative abundance of *Bifidobacteria* in the gut microbiome [123].

Probiotics are live microorganisms available in dietary sources that may exert beneficial effects on the host when ingested in adequate amounts, maintaining homeostasis in gut mucosa by enhancing integrity of the gut barrier, increasing the production of butyrate, and strengthening the TJ proteins (such as occludin and claudin-3) [124]. These substances benefit the host’s health by enhancing immunity through immunomodulation and by harmonizing the immune response. They modify the gut microbiota, promote colonization resistance, and suppress pathogens. Additionally, probiotics regulate inflammation-related cytokines, produce SCFAs, and regulate the expression of glucagon-like peptide-1 (GLP-1) [125]. Some studies and clinical trials support the hypothesis that the use of probiotics may play an important role in the regulation of the gut microbiota in adult patients with diabetes; specifically, *Lactobacillus* and *Bifidobacterium* strains may be effective in the prevention and management of the disease [126]. The role of probiotics in treating T1DM in children is poorly studied, however. There have been few trials, and the results of these trials are not completely conclusive in identifying an improvement in glycemic control. There was also no evidence of any changes in markers of inflammation associated with the administration of probiotics [127]. It was demonstrated that children with newly diagnosed T1DM can benefit from standard treatment combined with a high-potency, multi-strain probiotic preparation, Vivomixx^®^ (containing *Lactobacillus paracasei*, *Lactobacillus plantarum*, *Lactobacillus acidophilus*, *Lactobacillus delbruecckii* subsp. *bulgaricus*, *Bifidobacterium longum*, *Bifidobacterium infantis*, *Bifidobacterium breve* and *Streptococcus termophilus*) resulting in better glycemic control and a decrease in insulin requirements. Especially, results suggested a significant decrease in glycated hemoglobin HbA1c and a markedly greater number of children achieving disease remission in the treatment group [128,129]. Elsewhere, the findings of a rigorous double-blind, placebo-controlled, randomized trial suggested that the administration of probiotics *Lactobacillus rhamnosus GG* and *Bifidobacterium lactis BB12*, in a specific manner and dosage, did not have a significant impact on preserving the residual function of pancreatic β cells in children newly diagnosed with T1DM. Despite the potential of probiotics to offer various health benefits, this particular study suggested that, in the early stages of T1DM, these strains do not help maintain the functionality of pancreatic β cells, which are responsible for producing insulin [130].

Synbiotics consist of a combination of specific probiotic strain(s) with the prebiotics that feed them, they are created to improve the survival of probiotics in the gastrointestinal tract [131]. The consumption of synbiotics has been shown to alleviate intestinal dysbiosis, improving the health of T1DM patients. The combination of prebiotics and probiotics might also be more effective in glycemic control, compared to using probiotics alone [132,133]. Moreover, a symbiotic combination of *Lactobacillus salivarius* and prebiotic fibers could increase the production of SCFAs and reduce the expression of inflammatory cytokines in patients with T1DM, thus improving integrity of gut barrier by promoting the growth of epithelial cells and enhancing TJs between cells [119]. Conversely, recent research on T1DM children highlighted the lack of scientific evidence that the use of synbiotics causes any changes in glycemic or inflammatory control [127].

### 8.2. Faecal Microbiome Transplantation

The therapeutic strategy of FMT to treat or prevent T1DM is based on the possibility of correcting intestinal dysbiosis and modulating the immune response. FMT generally delivers faecal microbiota from a thoroughly screened healthy donor into the small intestine via an oral capsule or duodenal tube, but it can also enter the large intestine via an enema or colonoscopy. Some preclinical studies on rodent models have shown potential mechanisms by which gut microbiota transplantation from healthy donors can delay or prevent the onset of T1DM, altering the immune lymphocyte landscape and restoring intestinal and immune homeostasis. To date, only one pilot clinical trial and a few case reports using FMT in T1DM have been performed and these have suggested a clinical potential for FMT in adult human patients with T1DM, providing a base for developing (donor) FMT as a targeted treatment in humans [118,134,135]. 

Clinical data on the use of FMT in children with T1DM are still limited. Some small pilot studies have started to evaluate the safety and efficacy of FMT in this group of patients. These studies aim to determine whether FMT can improve glycemic control, reduce inflammatory markers, and influence disease progression [136]. He et al. have shown that, although the pre-transplant baseline glycemia was not identical between the two pediatric patients examined, the number of transplants and the time interval between transplants impacted clinical outcomes; multiple transplants at slightly longer intervals may have an “enhancing” effect in terms of glycemic improvement [137].

## 9. Conclusions

The complex interrelationship between T1DM and the microbiome highlights the vital necessity for an in-depth comprehension of microbial dysbiosis in the aetiology and progression of this autoimmune disease. A substantial body of evidence from numerous studies indicates that individuals with T1DM display notable alterations in both their gut and oral microbial compositions when compared to healthy individuals. This suggests that dysbiosis may play a pivotal role in the immune dysregulation observed in T1DM. It has been demonstrated that the gut microbiome exerts influence over the immune system via several mechanisms, including the modulation of gut permeability, the production of SCFAs, and interactions with the mucosal immune system. Dysbiosis of the gut microbiome can result in an imbalance of these processes, which may potentially trigger or exacerbate the autoimmune response that characterises T1DM. While it is also possible that oral dysbiosis may contribute to the development of systemic inflammation, which in turn may influence the progression of T1DM. 

Therefore, it is of great importance to study dysbiosis in both the gut and oral microbiomes for several reasons. Firstly, it may reveal novel biomarkers for the early diagnosis and progression-monitoring of T1DM. As previously stated, alterations in gut microbiome diversity occur before the onset of T1DM [13]. In this condition, a reduction in microbial diversity and an increase in Bacteroidetes are observed in most cases. The presence of this genus has been found to correlate with increased LPS levels, while its decrease is associated with lower metabolic endotoxemia and reduced inflammatory status [138]. Davis-Richardson et al. observed an increase in the abundance of *Bacteroides dorei*, which they suggest may serve as a potential indicator of diabetes risk in Finland [69]. However, it should be noted that the gut microbiome is subject to significant external influences, including geographic location, dietary habits, and hygiene practices. Consequently, the findings may not be directly applicable to other regions.

Secondly, an understanding of the specific microbial shifts associated with T1DM could facilitate the development of targeted therapeutic strategies, such as the use of prebiotics, probiotics, synbiotics, and FMT aimed at restoring healthy microbiomes. Finally, insights gained from microbiome research may contribute to the development of personalised medicine approaches, allowing for the implementation of tailored treatments that consider an individual’s unique microbial profiles. In conclusion, the study of microbiome dysbiosis in T1DM offers considerable potential for advancing our understanding of the disease and improving patient outcomes. Future research should focus on longitudinal studies to monitor changes in the microbiome over time, investigate the causal relationships between specific microbial species and T1DM, and explore the potential of microbiome-modulating therapies. By elucidating the complexities of the gut–oral-immune axis, we can pave the way for the development of novel strategies to prevent and manage T1DM, thereby enhancing the quality of life for those affected by this chronic condition.

## Figures and Tables

**Table 1:** Variations in bacterial abundance in the gut microbiome of individuals with T1DM in comparison to healthy individuals. **↑**—increased abundance, **↓**—decreased abundance.

Author	Ref.	Main Findings in T1DM Individuals
Devaraj et al. (2009)	[47]	**↑** Circulating LPS
Yuan et al. (2022)	[48]	**↑** Gene-related LPS biosynthesis
Traversi et al. (2017) Mejía-Leon et al. (2014)	[51,52]	Loss of α-diversity and β-diversity
Cinek et al. (2018) Leiva-Gea et al. (2018)	[58,60]	**↓** Firmicutes/Bacteroidetes ratio
Brown et al. (2011)	[67]	**↑** *Bacteroides*, *Clostridium, Veillonella, Ruminococcus, Escherichia, Streptococcus, Enterobacter*, *Lactobacillus* **↓** *Prevotella, Akkermansia, Bifidobacterium*, *Roseburia, Faecalibacterium, Eubacterium*
Giongo et al. (2011)	[53]	**↑** *Bacteroides ovatus*, *B. thetaiotaomicron*, *B. uniformis* **↓** *B. fragilis*, *B. vulgatus*
Davis-Richardson et al. (2014)	[69]	**↑** *Bacteroides dorei* and *vulgatus*
Matos et al. (2021)	[70]	Some *Bacteroides dorei* strains invade and damage intestinal epithelial TJs
Vatanen et al. (2018) TEDDY Study	[72]	No significant taxonomic differences in gut microbiota between T1DM patients and controls
Biassoni et al. (2020)	[76]	**↑** *Bacteroides stercoris*
Endesfelder et al. (2016)	[79]	Presence of three bacterial communities prior to the development of islet autoantibodies

**Table 2:** Variations in bacterial abundance in the oral microbiome of individuals with T1DM in comparison to healthy individuals. **↑**—increased abundance, **↓**—decreased abundance.

Author	Ref.	Main Findings in T1DM Individuals
Vila et al. (2019)	[96]	**↓** saliva **↑** pathogenic and acidogenic bacteria (*Streptococcus mutans* and *Lactobacillus*)
Torrungruang et al. (2015)	[97]	**↑** periodontopathogenic bacteria (*Porphyromonas gingivalis* and *Aggregatibacter actinomycetemcomitans*)
Chen et al. (2020)	[99]	**↑** *Streptococcus mutans*, *Lactobacillus*, and *Actinomyces*
de Groot et al. (2017)	[101]	**↑** Actinobacteria and Firmicutes (*Streptococcus* spp., *Actinomyces* spp., *Rothia* spp.) **↓** Bacteroidetes and Proteobacteria
Moskovitz et al. (2021)	[104]	**↑** *Streptococcus* genus **↓** *Mogibacterium* genus
Carelli et al. (2023) Silvestre et al. (2009)	[106] [107]	**↑** *S. mitis, S. oralis, S. anginosus, S. gordonii*
Yuan et al. (2022)	[108]	**↑** opportunistic pathogens (*Streptococcus*, *Granulicatella*, *Rothia* and *Rhodococcus*) **↓** *Veillonella* and *Prevotella*
Kunath et al. (2022)	[109]	**↑** *S. mutans* **↓** *S. salivarius*
